# Phenotypic differentiation in love song traits among sibling species of the *Lutzomyia longipalpis* complex in Brazil

**DOI:** 10.1186/s13071-015-0900-8

**Published:** 2015-05-28

**Authors:** Felipe M. Vigoder, Nataly A. Souza, Reginaldo P. Brazil, Rafaela V. Bruno, Pietra L. Costa, Michael G. Ritchie, Louis B. Klaczko, Alexandre A. Peixoto

**Affiliations:** Laboratório de Genômica Evolutiva, Departamento de Genética, Universidade Federal do Rio de Janeiro, Rio de Janeiro, Brazil; Laboratório Interdisciplinar de Vigilância Entomológica em Diptera e Hemiptera, Instituto Oswaldo Cruz, FIOCRUZ, Rio de Janeiro, Brazil; Laboratório de Doenças Parasitárias, Instituto Oswaldo Cruz, FIOCRUZ, Rio de Janeiro, Brazil; Laboratório de Biologia Molecular de Insetos, Instituto Oswaldo Cruz, FIOCRUZ, Rio de Janeiro, Brazil; Instituto Nacional de Ciência e Tecnologia em Entomologia Molecular/CNPq, Rio de Janeiro, Brazil; Departamento Imunologia, Centro de Pesquisas Aggeu Magalhães-Fiocruz, Recife, Pernambuco Brazil; School of Biology, University of St Andrews, St Andrews, UK; Departamento de Genética, Evolução e Bioagentes, Instituto de Biologia, Universidade Estadual de Campinas, UNICAMP, Campinas, São Paulo Brazil

**Keywords:** Sexual behaviour, Sand fly, Copulation song, Copulatory courtship, Insect vector, Species complex

## Abstract

**Background:**

Brazilian populations of *Lutzomyia longipalpis* may constitute a complex of cryptic species, and this report investigates the distribution and number of potential sibling species. One of the main differences observed among Brazilian populations is the type of acoustic signal produced by males during copulation. These copulation song differences seem to be evolving faster than neutral molecular markers and have been suggested to contribute to insemination failure observed in crosses between these sibling species. In previous studies, two main types of copulation songs were found, burst-type and pulse-type. The latter type can, in turn, be further subdivided into five different patterns.

**Methods:**

We recorded male song from 13 new populations of the *L. longipalpis* complex from Brazil and compared the songs with 12 already available.

**Results:**

Out of these 25 populations, 16 produce burst-type and 9 produce pulse-type songs. We performed a principal component analysis in these two main groups separately and an additional discriminant analysis in the pulse-type group. The pulse-type populations showed a clear separation between the five known patterns with a high correspondence of individuals to their correct group, confirming the differentiation between them. The distinctiveness of the burst-type subgroups was much lower than that observed among the pulse-type groups and no clear population structure was observed. This suggests that the burst-type populations represent a single species.

**Conclusion:**

Overall, our results are consistent with the existence in Brazil of at least six species of the *L. longipalpis* complex, one with a wide distribution comprising all the populations with burst-type songs, and five more closely related allopatric siblings with different pulse-type song patterns and more restricted distribution ranges.

**Electronic supplementary material:**

The online version of this article (doi:10.1186/s13071-015-0900-8) contains supplementary material, which is available to authorized users.

## Background

Understanding speciation is one of the central questions in evolutionary biology. Many authors consider sexual selection to be one of the main causes of speciation, creating reproductive barriers that can prevent gene flow [1, 2]. Acoustic communication has been implicated in sexual selection and can act as a recognition signal in many animals, from insects to primates, with sibling species showing distinct songs [3–8]. Hence song variation could represent an important phenotype for understanding patterns of speciation in species complexes.

*Lutzomyia longipalpis* (Diptera: Psychodidae) males produce a song to females during copulation that may contribute to reproductive success [9–11]. This sand fly is the main vector of American visceral leishmaniasis and it constitutes a species complex [12–14]. However, the distribution and number of sibling species is still unclear, particularly in Brazil [11, 15, 16]. Among the evidences for the existence of cryptic species in Brazil is the reproductive isolation observed in laboratory crosses between some populations [12, 17, 18] and the fact that they have differences in phenotypic characters, such as male copulation songs and sex pheromones, and moderate to high levels of genetic divergence in some molecular markers such as microsatellites and several protein coding genes, including some associated with sexual behaviour in *Drosophila* [11, 13, 16, 18–30].

The reproductive isolation observed between Brazilian populations of the *L. longipalpis* complex is caused by insemination failure during copulation in crosses between only certain populations [13, 17, 18]. This suggests that after copulation has started, there is signalling that is important for insemination to occur. Because males sing during copulation and the insemination failure is observed in crosses between populations producing different types of copulation songs, this acoustic signal might have a role in species recognition, acting as a reproductive barrier reducing gene flow and potentially preventing gamete wastage [10, 11].

The different cryptic species of the *L. longipalpis* complex can be separated into two main groups according to the types of copulation song which males produce: pulse-type and burst-type [10, 11]. The Burst-type song is composed of trains with highly polycyclic pulses (“bursts”) modulated in frequency and amplitude. The pulse-type group on the other hand, has previously been shown to include five different song patterns designated as P1, P2, P3, P4 and P5 [10, 11].

Here we test the variability of song amongst *L. longipalpis* using a more comprehensive geographic sampling analysing 13 new Brazilian populations and comparing them with 12 populations analysed by Araki et al. [11]. We examine the number of phenotypic song types found within each of the pulse- and burst-type song patterns, specifically asking if the pulse-type group represents multiple species and the burst-type only one. Overall, our results are consistent with the existence of at least six species of the *L. longipalpis* complex in Brazil, one with a wide distribution comprising all the populations with burst-type songs despite some level of geographic structuring, and five more closely related allopatric siblings with different pulse-type song patterns and more restricted distribution ranges.

## Methods

Sand flies were collected using CDC light traps and the *L. longipalpis* individuals were identified according to Young & Duncan [31]. Samples were obtained from the following localities: Afonso Claudio (September 2009) (20°04'S, 41°08'W) (Espírito Santo State); Ipanema (March 2010) (19°48′S, 41°42′ W), Nova Porteirinha (December 2007) (15°48′S, 43°18′W) and Lassance (January 2009) (17°53′S, 44°34′W) (Minas Gerais State); Pirenópolis (December 2007) (15°51′S, 48°57′W) (Goiás State); Aracajú (October 2010) (10°54′S, 37°4′W) (Sergipe State); Itamaracá (September 2007) (7°45′S, 34°51′W) and Passira (December 2007) (7°56′S, 35°35′W) (Pernambuco State); Barcarena (December 2007) (1°31′S, 48°37′W), Cametá (February 2010) (2°15′S, 49°30′W) and Camará (June 2010) (2°25′S, 54°43′W) (Pará State); and Palmas (July 2006 and April 2010) (10°10′S, 48°19′W) (Tocantins State).

*Lutzomyia longipalpis* males show a polymorphism in the number of abdominal spots, which are sex pheromone glands. Males can have either one pair in the fourth tergite (called 1S) or two pairs in the third and fourth tergites (called 2S). The second pair in the third tergite can also be smaller than the pair in the fourth and this is sometimes called the intermediate form [13, 17]. Although the spot polymorphism usually has no taxonomical value [13], in localities where sympatric species producing either pulse-type or burst-type song are found, males can also be differentiated by the number of spots and in these cases individuals with an intermediate spot phenotype are rare [10, 11, 20, 21, 24]. Of the new samples analysed in the present work we observed 1S and 2S males in sympatry only in the locality of Palmas, therefore this sample was subdivided into Palmas 1S and Palmas 2S.

The recordings were made in accordance with Souza et al. [10] using a male and a female in each trial. Insects were placed in a small chamber inside an INSECTAVOX [32], which contains the microphone. Trials were also filmed using a Sony Hi8CCD-TRV65 video camera. Both sound and video were recorded using a Panasonic DMR-ES10 DVD recorder. All trials were performed at 25 °C ± 1 °C and if no copulation occurred in 4–5 min the couple was replaced. Most recordings were performed using either wild caught sand flies or their F1 progeny.

After recording, songs were digitized using CED 1401 and analysed using Spike 2 software (version 4.08), both from Cambridge Electronic Design, United Kingdom. The parameters measured were: inter-pulse or inter-burst interval (IPI/IBI), number of pulses or bursts per train (NP/NB), train length (TL) and the carrier frequency of the pulse train (Freq). In the pulse-type populations (see Results) we also measured the cycles per pulse (CPP) and the proportion of pulses with alternated amplitudes (AmpAlt), which is the proportion of pulses that have amplitudes either higher or lower than both adjacent pulses. The alternated amplitude analysis was also performed in the populations previously described by Souza et al. [10] and Araki et al. [11]. Note that in the previous analysis of AmpAlt carried out in Araki et al. [11] for a couple of populations, this song parameter was computed based on a simple visual inspection of the song traces and small differences in pulse amplitude were therefore ignored. In the current analysis this was carried out automatically based on exact amplitude values.

Statistical analysis was performed using SPSS version 19 software. All statistical analyses were done using the new populations reported here combined with data from the populations analysed by Souza et al. [10] and Araki et al. [11].

## Results

Males from all the new samples produced song during copulation, i.e., after they grabbed the female with their genital clamp. The copulation songs showed the same characteristics described previously for *L. longipalpis*, with primary and secondary songs [10, 11]. The analysis here concentrates on the primary song as it shows more variation among populations with a clear and consistent pattern and is produced in every copulation, while the secondary song is a long train of song with an uneven distribution of polycyclic pulses not produced by every male (see Araki et al. [11]). Also even when it is produced, the secondary song is similar in all populations.

**Fig. 1:**
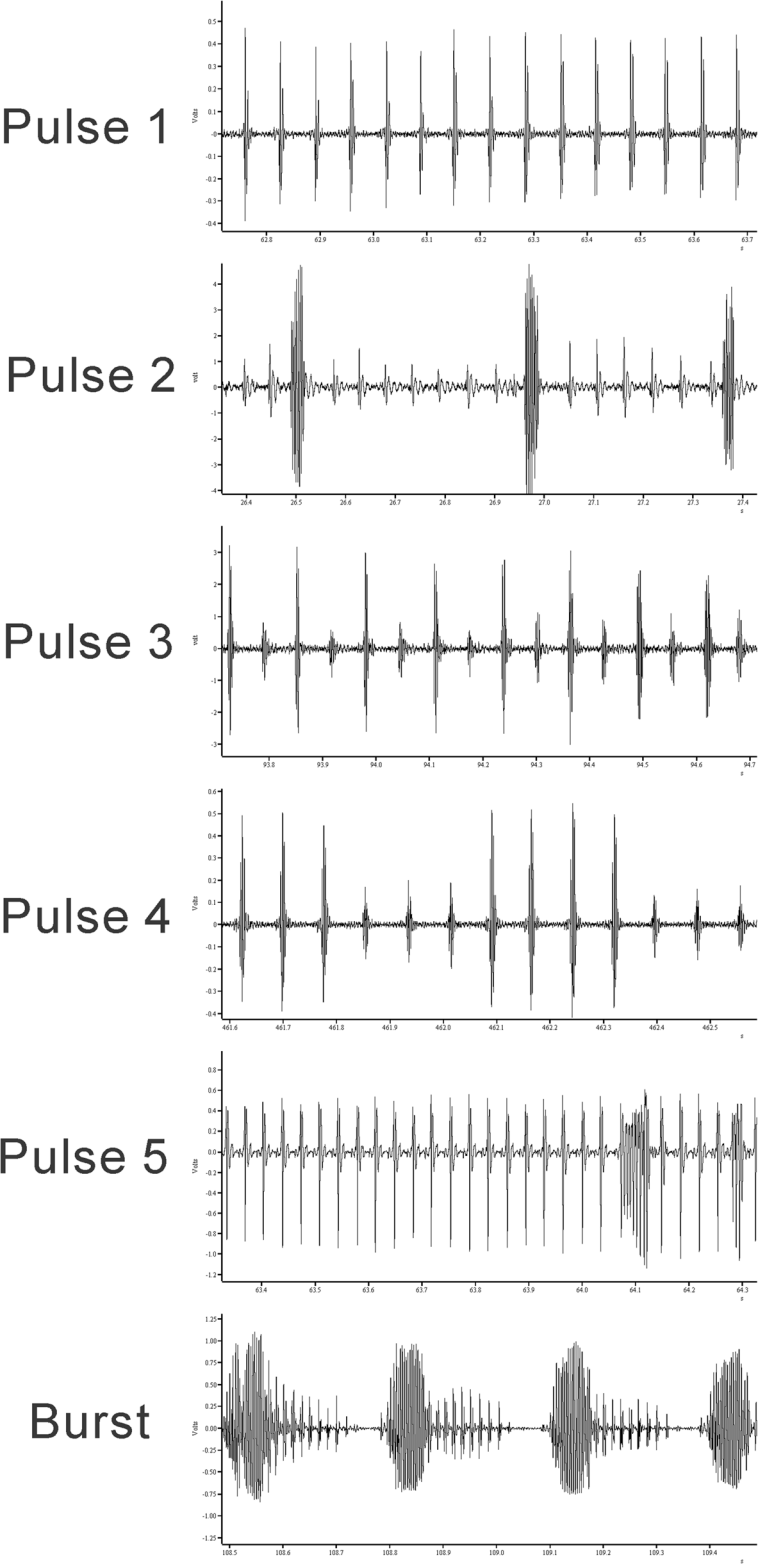
Examples of the song traces of all 6 patterns observed in Brazilian populations of *L. longipalpis* (see text for further details). The first five correspond to the different pulse-type patterns or subtypes and the last one to the burst-type

Figure 1 shows examples of traces of the different song types and patterns observed in *L. longipalpis* populations based on the current and previously published data. Audio samples of each song type are available in the online supplementary material (Additional files 1, 2, 3, 4, 5 and 6). The songs are consistent in each population with every male producing a similar pattern. Because burst-type and pulse-type songs are clearly distinguishable both by visual inspection (Fig. 1) and sound (Additional files 1, 2, 3, 4, 5 and 6) and do not share the same descriptive parameters (see Methods), the analyses were performed for each of these groups separately. It should be noted that in Palmas the 2 sympatric populations have a different type of song.

### Pulse-type populations

The P1 songs are composed of trains of similar pulses with usually two or three cycles per pulse. P2 songs differ from P1 mainly by the presence of interspersed polycyclic pulses between nearly monocyclic pulses. The P3 songs are characterized by an almost perfect alternation of high and low amplitude pulses. P4 also presents a modulation in pulse amplitude but this occurs throughout the train with a more gradual amplitude oscillation or with alternating sequences of 2 or more high and low amplitude pulses. Finally, P5 is characterized by having a very short inter-pulse interval (~35 ms while other Pulse songs have > 50 ms) with some very polycyclic pulses in the end of the train (Fig. 1).

**Fig. 2:**
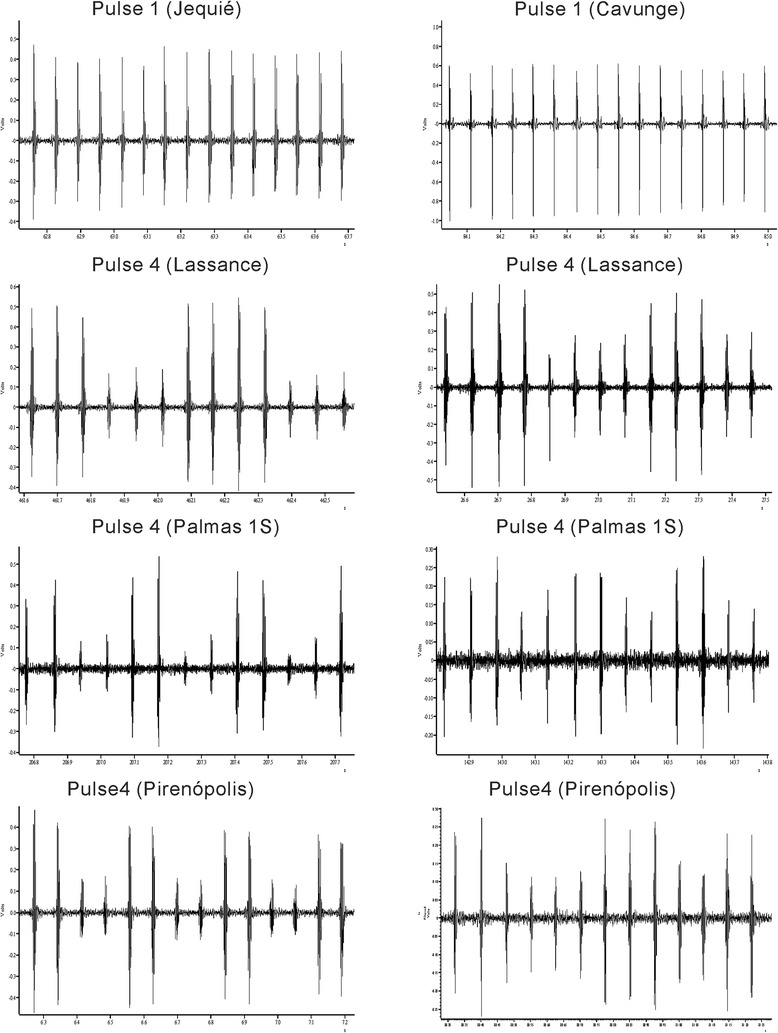
Examples of song traces from pulse-type populations of *L. longipalpis* from the localities of Lassance, Pirenópolis and Palmas 1S. The figure shows ~1 s of song in each case. Two traces from the populations Lassance, Pirenópolis and Palmas 1S are shown to illustrate the variation in the P4 pattern

Among the 13 new samples, males from the populations of Pirenópolis, Lassance and Palmas 1S produced Pulse-type songs. These 3 populations produce the P4 pattern (Fig. 2, see also Fig. 1). Table 1 shows the mean values of the different song parameters for each population. Values for most song parameters, except AmpAlt, from the populations of Jacobina, Lapinha, Sobral 1S, Teresina, Jaiba 1S and Estrela 1S have been published before by Souza et al. [10] and Araki et al. [11] but were included here for comparison. All values of AmpAlt were obtained in the present work.

**Table 1:** Mean (±SE) values of all parameter analysed in the pulse-type populations and their respective pattern

	N	Type	IPI (ms)	NP	TL (s)	Freq (Hz)	CPP	AmpAlt (%)
Jacobina	11	Pulse 1	51.80 (±1.60)	44.90 (±3.00)	2.30 (±0.20)	231.90 (±11.00)	2.60 (±0.20)	0.60 (±0.08)
Lapinha	15	Pulse 2	57.30 (±1.30)	58.10 (±2.80)	3.30 (±0.20)	284.10 (±6.90)	1.40 (±0.10)	0.64 (±0.07)
Sobral 1S	11	Pulse 3	65.60 (±1.00)	32.30 (±1.00)	2.10 (±0.1)	306.50 (±5.80)	3.10 (±0.10)	0.93 (±0.05)
Teresina	7	Pulse 3	65.50 (±0.74)	32.81 (±2.75)	2.08 (±0.18)	298.67 (±9.05)	3.11 (±0.15)	0.90 (±0.13)
Jaiba 1S	4	Pulse 4	66.68 (±4.12)	33.00 (±6.81)	2.10 (±0.36)	298.38 (±7.67)	2.81 (±0.33)	0.54 (±0.05)
Pirenópolis	9	Pulse 4	74.47 (±1.26)	34.06 (±1.85)	2.46 (±0.16)	288.80 (±10.74)	3.28 (±0.12)	0.54 (±0.05)
Palmas 1S	4	Pulse 4	71.68 (±5.65)	29.28 (±3.69)	1.99 (±0.10)	295.87 (±8.89)	2.91 (±0.06)	0.52 (±0.02)
Lassance	7	Pulse 4	69.40 (±2.54)	38.02 (±2.24)	2.58 (±0.20)	288.70 (±3.54)	3.05 (±0.09)	0.54 (±0.05)
Estrela 1S	5	Pulse 5	36.59 (±0.89)	101.30 (±4.23)	3.66 (±0.08)	174.05 (±0.43)	1.99 (±0.16)	0.67 (±0.04)

*N* number of samples; *IPI* inter-pulse interval; *NP* number of pulses per train; *TL* train length; *Freq* carrier frequency; *CPP*: cycles per pulse; *AmpAlt* the proportion of pulse that have amplitudes either higher or lower than both adjacent pulses. The values of *IPI*, *NP TL Freq* and *CPP* of the population of Jacobina, Lapinha, Sobral 1S, Teresina, Jaiba 1S and Estrela 1S have been published previously by Souza, et al. [10] and Araki, et al. [11]

**Fig. 3:**
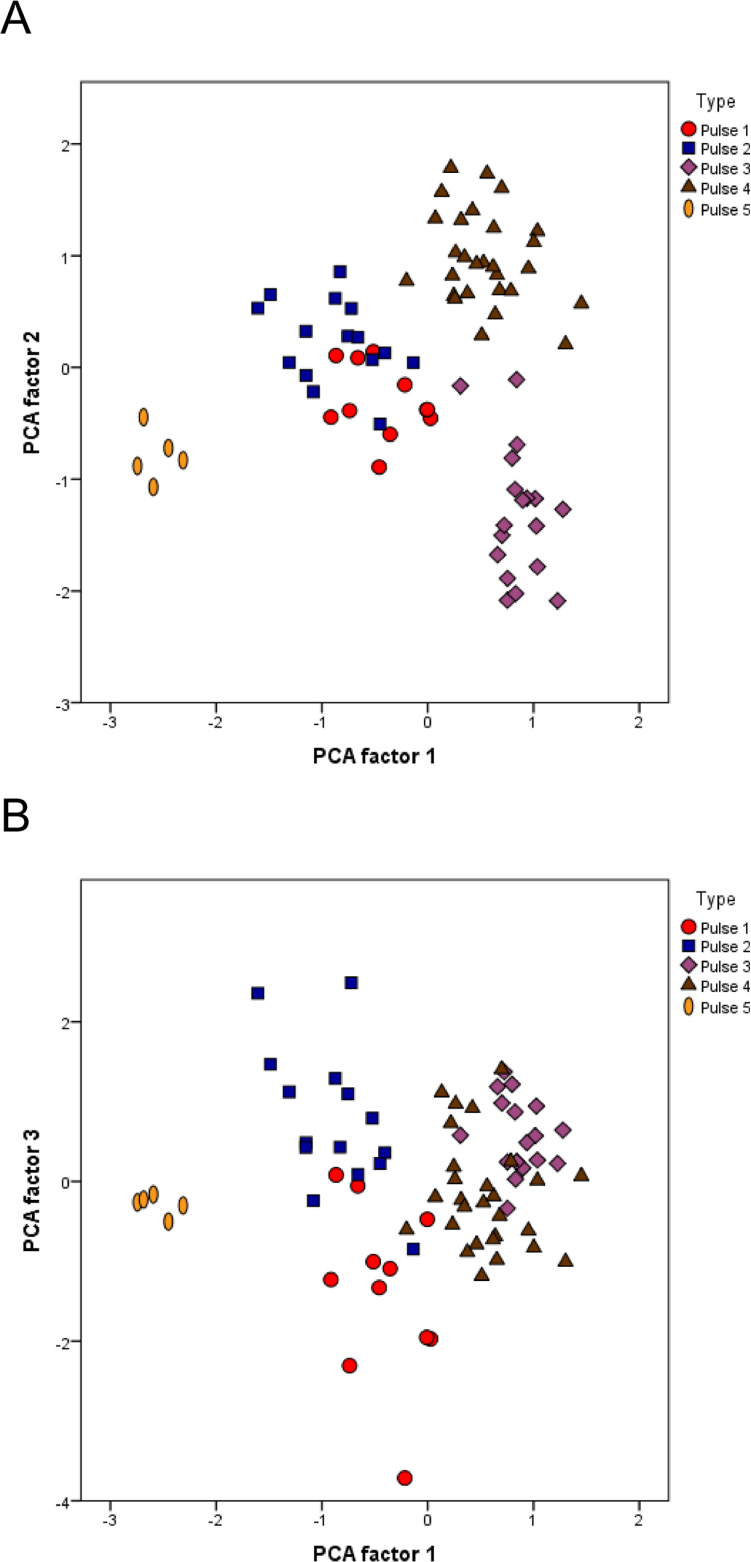
Scatter-plots of the first 3 factors obtained in the Principal Component Analysis of the pulse-type populations. (**a**) shows the plot of the first 2 canonical functions, (**b**) shows the plot of the first and third canonical functions

A principal component analysis (PCA) was made using the six parameters (See above). PCA is a multivariate statistical test that transforms the variables creating new factors focusing on where the main variance in the data is and is performed independently of any assumptions about groups present in the data. The first 3 factors accounted for 83 % of the total variance (51 %, 18 % and 14 % respectively). With the exception of AmpAlt, all parameters had a similar influence to the first factor with NP and TL having an inversed influence (Table 2). The 3 factors showed significant differences when comparing the populations grouped according to the pulse pattern with an ANOVA test (p < 0.001) and the 5 groups form clear clusters when the factors are plotted (Fig. 3a and b). Although P1 & P2 overlap in plots of PCA1 vs 2, they are distinct in PCA 2 vs. 3 (Fig. 3a and b). In order to quantitatively assess the distinctness of the clusters in song type, we also performed secondary analysis using the pulse type as *a priori* groups for a standard discriminant analysis (DCA) using the default configuration of SPSS. In the DCA the multiple variables are used to find the best model that maximises variance in the data between groups, and allocation tests for each individual (omitting them from the original classification) allows quantification of how accurately group membership can be determined.

**Table 2:** Loadings of the principal components analysis in the pulse-type populations

Variable	PC 1	PC 2	PC3
IPI	.785	.406	.291
NP	-.938	-.007	.224
TL	-.715	.343	.576
Freq	.726	.122	.448
CPP	.703	.034	-.037
AmpAlt	.223	-.865	.425

**Fig. 4:**
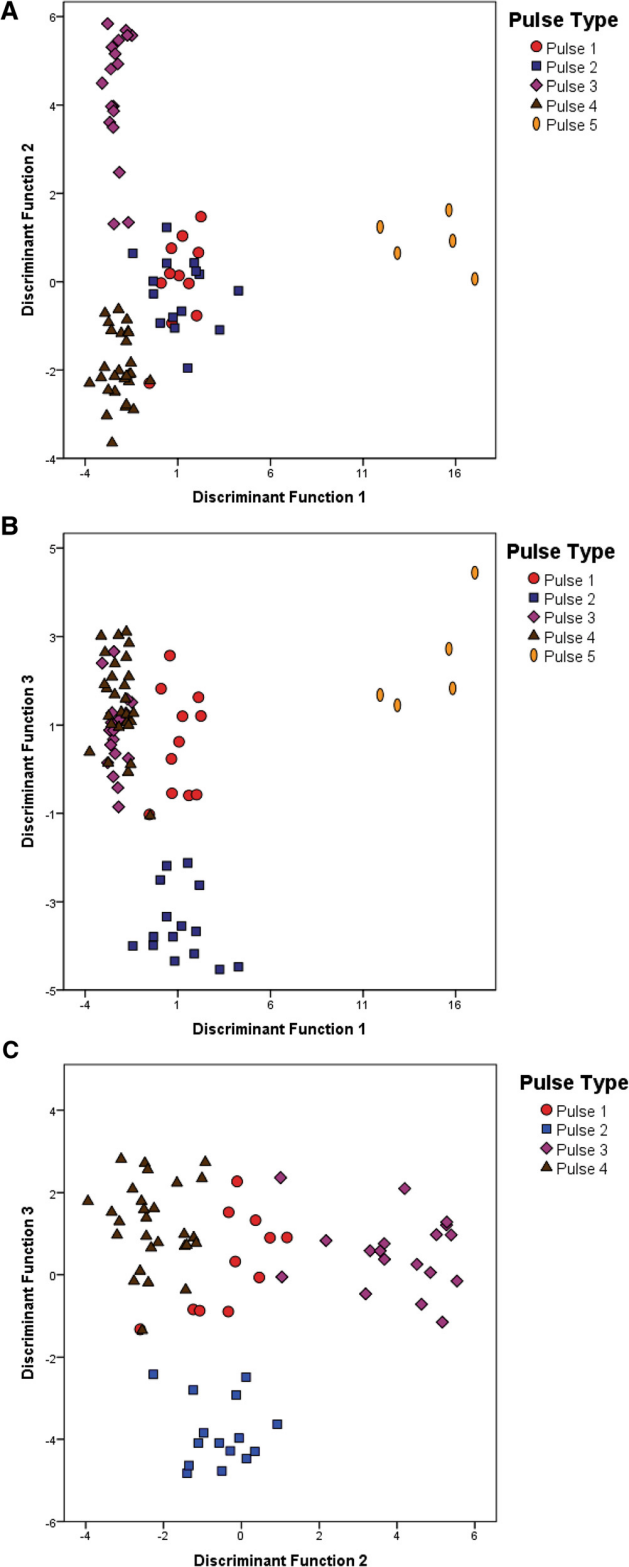
Scatter-plots of the first 3 canonical functions obtained in the discriminant analysis of the pulse-type populations. (**a**) shows the plot of the first 2 canonical functions, (**b**) shows the plot of the first and third canonical functions and (**c**) the plot of the second and third canonical functions. P5 was not included in **c** since it forms a clear cluster in (**a**) and (**b**) and it helps visualise the differences among the other 4 song types

The first three discriminant canonical functions explained 94 % of the total variation (61 % and 20 % and 13 % respectively). NP, AmpAlt and CPP had the highest load on each of the discriminant functions, which means they are the parameters creating most of the differences between the groups. All groups formed clear clusters with an almost perfect separation as can be seen in the scatter plots of the first three canonical functions (Figs. 4a and 4b). Plotting the first 2 functions showed a clear separation of P3 and P4 (Fig. 4a) and plotting the first and third function P1 and P2 becomes distinguished (Fig. 4b). Figure 4c also shows a plot of the second and third canonical functions but P5 was not included since it forms an obvious cluster in the other 2 figures and in the present plot it has a scatter distribution. Figure 4c shows a more clear separation of P1, P2, P3 and P4.

The five groups had a perfect 100 % correct assignment of individuals to each original group, confirming that the five song types are quantitatively completely distinct.

### Burst-type populations

**Fig. 5:**
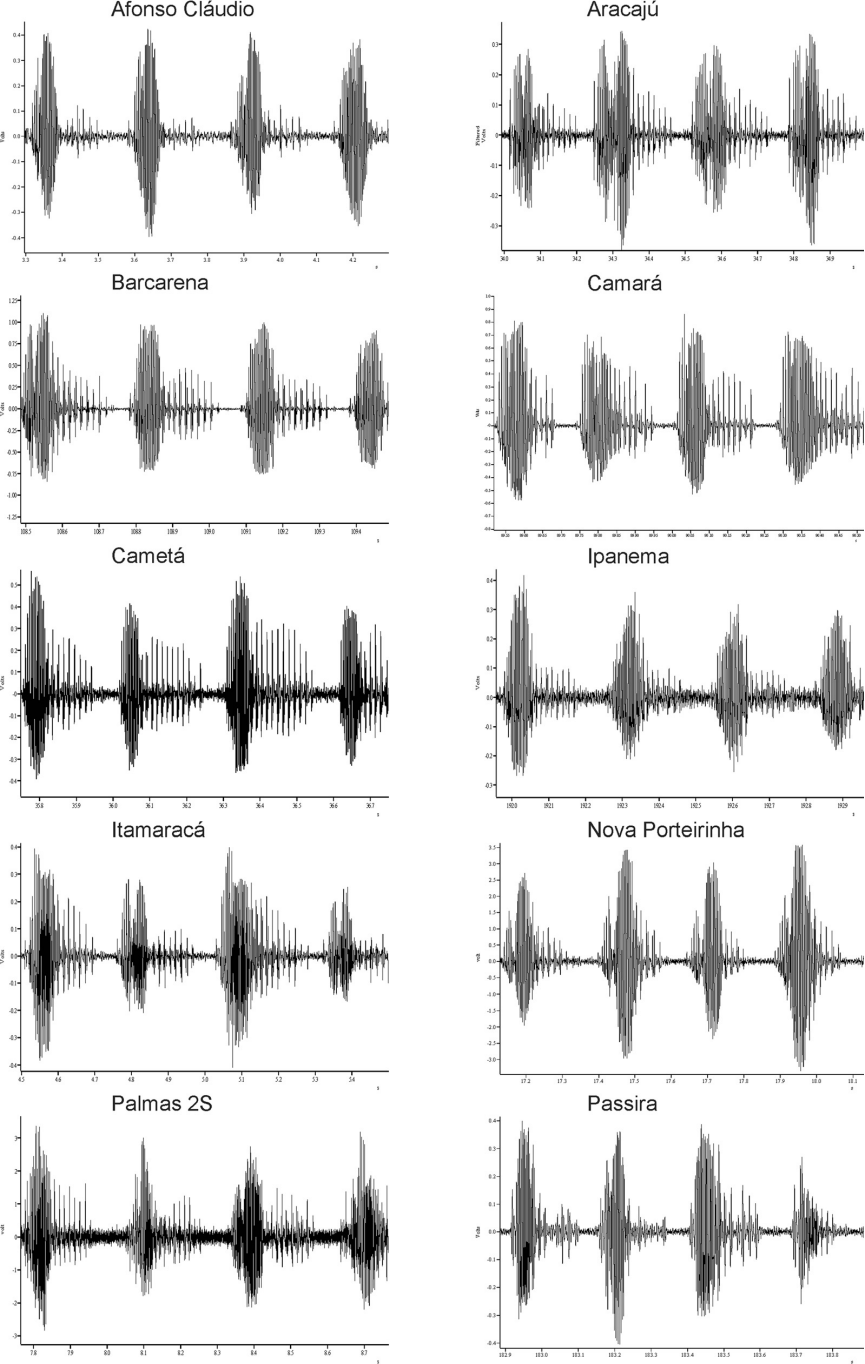
Examples of the song traces from the burst-type populations *L. longipalpis* from the localities of Afonso Cláudio, Aracajú, Palmas 2S, Ipanema, Barcarena, Cametá, Camará, Itamaracá, Passira and Nova Porteirinha. The figure shows ~1 s of song in each case

Males from Afonso Cláudio, Aracajú, Palmas 2S, Ipanema, Barcarena, Cametá, Camará, Itamaracá, Passira and Nova Porteirinha produced burst-type songs (Fig. 5, see also Fig. 1). No clear song pattern variation was observed within this group. Table 3 shows the mean values of the different song parameters for each population. The values of the burst-type populations obtained by Souza et al. [10] and Araki et al. [11] were not included in the table because no new measurement of song parameters were carried out in those populations.

**Table 3:** Mean (±SE) values of all parameter analysed in the burst-type populations

	N	IPI/IBI (ms)	NP/NB	TL (s)	Freq (Hz)
Nova Porteirinha	5	243.51 (±5.61)	11.37 (±0.99)	2.62 (±0.23)	263.67 (±6.18)
Barcarena	11	255.15 (±6.46)	8.64 (±1.12)	2.16 (±0.29)	278.85 (±3.79)
Itamaracá	6	264.20 (±7.75)	11.97 (±1.73)	3.05 (±0.43)	286.36 (±9.17)
Passira	6	249.73 (±14.20)	11.04 (±0.74)	2.69 (±0.29)	272.06 (±5.66)
Camará	7	241.36 (±9.72)	11.64 (±1.23)	2.74 (±0.31)	268.17 (±5.14)
Palmas 2S	3	283.15 (±28.78)	11.42 (±1.45)	3.10 (±0.33)	276.34 (±2.90)
Afonso Cláudio	2	266.73 (±20.95)	14.25 (±2.75)	3.65 (±0.42)	255.00 (±5.43)
Aracajú	12	269.78 (±12.71)	11.77 (±0.80)	3.06 (±0.27)	289.06 (±6.01)
Cametá	6	256.93 (±23.02)	9.92 (±1.23)	2.40 (±0.36)	271.29 (±7.42)
Ipanema	8	243.71 (±12.79)	15.02 (±0.69)	3.57 (±0.28)	285.30 (±6.17)

*N* number of samples; *IBI* inter-burst interval; *NB* number of bursts per train; *TL* train length; *Freq* carrier frequency

**Fig. 6:**
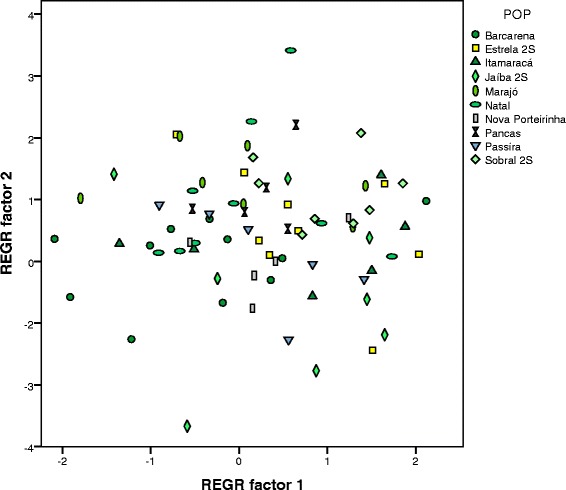
Scatter-plots of the first 2 factors obtained in the Principal Component analysis of the burst-type populations

The principal component analysis showed that the first two factors represented 83.6 % of the total variance (47.5 % and 36.1 % respectively) and the values obtained are plotted in Fig. 6. NB and TL had a strong influence on the first factor and IBI and Freq a strong inverse influence (Table 4). The ANOVA performed with the principal component factors showed no significant difference for the first factor among all populations (p = 0.14) but significant differences in the second one (p = 0.002). However, a post-hoc analysis of the second factor using Bonferroni correction showed that only Jaíba 2S had a significant difference to other populations, Sobral 2S and Marajó (p < 0.05) (Additional file 7). The fact that the vast majority of the pairwise comparison did not show any difference suggests a lack of population structure in this group.

**Table 4:** Loadings of the principal components analysis in the burst-type populations

Variable	PC 1	PC 2
IBI	.222	-.836
NP	.925	.336
TL	.991	-.042
Freq	-.106	.794

## Discussion

The occurrence of cryptic species is common among insects and very often they can be separated by phenotypic traits involved in mating [33]. In *L. longipalpis* sensu lato, the male song produced during copulation clearly suggests the existence of cryptic species within this taxon. The different song patterns observed in Brazilian populations suggests that song is evolving faster than other phenotypic traits, probably because it is under sexual selection [2, 3, 5, 34]. Characteristics under sexual selection tend to evolve faster than other traits making them good markers to differentiate closely related species [35, 36]. Acoustic communication has often been implicated in sexual selection [6] and this is likely that it has this role in the *L. longipalpis* species complex since the songs are produced during copulation and some studies suggest the existence of a mechanism of recognition during mating that is important for insemination success [13, 18].

**Fig. 7:**
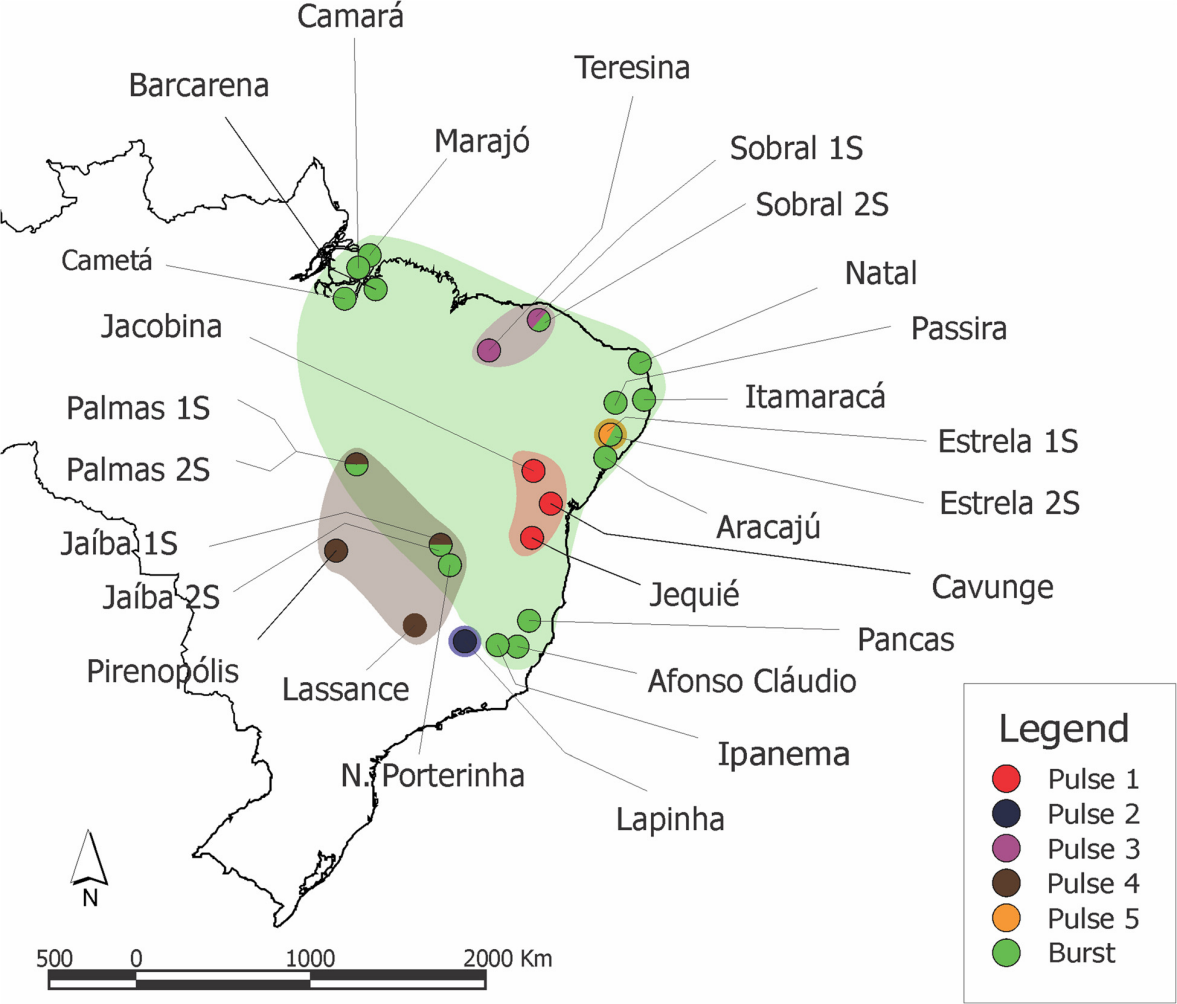
Map of Brazil showing the approximate geographical localization of all *L. longipalpis* populations that had their male copulations songs analysed so far (see text for further details). The colours of the circles symbolize the song pattern found in each population. The lighter patch of similar colour in the background marks the hypothetical distribution area of each cryptic species

Our analysis of the song produced by males from a number of Brazilian populations of *L. longipalpis* s.l. shows the existence of two main groups within this species complex, one producing burst-type songs and the other producing pulse-type songs (Fig. 7). The differences between the two song types are so large that it is difficult to quantitatively compare them [10, 11]. A third type is called the “mix-type”, because it shares some features of burst- and pulse-type songs, and seems to be quite rare as it was only found in a single small sample so far by Araki et al. [11]. It is important to note, however, that the mix-type songs is a completely different pattern and no song observed outside of the Mesquita populations can be classified as such so far [11].

Until now, 9 populations have been found to produce five distinct patterns of pulse-type songs (Fig. 7). These five patterns or subtypes not only have qualitative differences, but the discriminant analysis also demonstrates quantitative variation that allows high discrimination among them (Fig. 4). These five groups also show geographical separation and no overlap between their distributions have yet been observed (Fig. 7).

Statistical analysis showed clear differentiation among the five groups and the discriminant function analysis found that 100 % of the individuals could be assigned to the correct group based on song pattern. P2 song type was found so far in a single locality (Lapinha Cava) in south-eastern Brazil (Fig. 7) and the P1 song is present only in the north-eastern state of Bahia (Fig. 7). P5 was also found in a single locality (Estrela de Alagoas) in the Northeast region, in sympatry with a burst-type population in (Fig. 7).

Consistent with the available molecular data [11, 25], the song analysis also suggests that P3 and P4 are the two closest related groups among the pulse-type groups, since they both have pulse amplitude variation being differentiated mainly by the type of this variation seen, either an almost perfect alternation between high and low amplitude pulses (P3) or a more continuous variation throughout the train (P4) (Fig. 2) and also having more similar values in most parameters (Table 1). Such amplitude variation is extremely unusual in dipteran song, but presumably could be detected by females as song is always produced in copula. The currently known distribution places P3 populations in the far northern part of north-eastern Brazil while P4 is found in the more central part of Brazil (Fig. 7).

Further investigation is necessary to confirm that all five different pulse-type patterns represent distinct species and if the songs are associated with reproductive isolation among them, especially as they are allopatric in nature. The divergence in song patterns observed among the different pulse-type *L. longipalpis* populations is similar to the variation seen among some closely related *Drosophila* species [37, 38], so it is quite possible that they represent distinct species. In addition, populations with different song types also produce different pheromones [11, 23] and crossing experiments have found reproductive isolation between at least two of these populations: Jacobina (P1) and Lapinha (P2) [18]. Moreover pulse-type populations with different patterns tend to have a considerable level of genetic divergence [11, 16, 17, 22].

Some small differences were also observed amongst the burst-type populations, but not close to the same extent as the Pulse-type populations. This group has a much wider geographical distribution in Brazil, ranging from the Southeast region up to the North region, crossing several different ecosystems (Fig. 7). Despite the similar song pattern, the statistical analysis of the copulation song of this group showed a difference between Jaíba 2S and a pair of other population. This difference could be an indication of the beginning of a separation of the Jaíba 2S population from the other Burst-type populations. However, the fact that the other 7 populations did not have significant differences suggests that even if it were true, the separation would be very recent and is qualitatively much less significant than that seen among pulse populations.

It is important to note that crossing experiments using different populations with burst-type songs showed they have normal insemination rates [13]. Finally, all burst-type populations that have had their sex pheromone analysed produced the same compound (cembrene) [11]. They have an overall smaller mean Fst (a widely used measure of genetic differentiation) [39, 40] of ~0.16 in the *per* gene in comparison among them [11, 13, 23] in contrast to the mean Fst observed among pulse-type populations, ~0.26 [11]. All this strongly suggests that the burst-type group constitute a single species.

In Palmas, as previously observed for the localities of Sobral, Jaíba and Estrela [10, 11], males of two sympatric species can be distinguished by the spot phenotype, 1S or 2S. Palmas 1S males produce pulse-type songs (P4) while Palmas 2S males produce burst-type songs. The occurrence of sympatric species in many localities is one of the strongest pieces of evidence that *L. longipalpis* is a species complex in Brazil. It also suggests the absence of very large ecological differences between the sibling species, reinforcing the idea that sexual selection is acting as the primary force of speciation in the *L. longipalpis* complex [11].

A striking aspect of the geographic distribution of the *L. longipalpis* complex is the large distribution of the burst-type species coupled with a low level of phenotypic variation and the restricted distribution and high level of variation among the pulse-type populations (Fig. 7). What could cause these contrasting patterns of differentiation between the two song-type groups? Multilocus coalescent analysis estimates a recent separation between the burst- and pulse types around 0.5 mya [26], so if the differences between pulse-types have evolved since then, the evolution of these song types has been extremely rapid. The biogeographic history of the region is poorly understood, but it is possible that after an initial separation between burst- and pulse populations the ecological and climatic changes during the Quaternary could have caused vicariance of the pulse-type populations in refugia with more stable climatic zones [41], whereas the burst-type was confined to a single refugium with a more rapid expansion following climatic amelioration. If the pulse-types had a more complex history of secondary contacts between developing forms [42], there would have been more potential for character displacement to influence song and pheromone divergence. More biological reasons for greater divergence could include greater dispersal and gene flow among the burst-type or higher effective population sizes. If sexual selection is stronger in the pulse-type due to mating system variation then mating signals may diverge more quickly, leading to greater levels of sexual isolation among species. Little is known about mating rates or variance in mating success in natural populations of these species, but some of these intriguing hypotheses could be investigated by analyses of broader patterns of genomic divergence between the forms [43].

## Conclusion

Our results show clear qualitative and quantitative variation among the male song patterns found in Brazilian populations of the *L. longipalpis* complex. These results confirm that the acoustic signals are a very good marker to differentiate these potential cryptic species. Six song patterns were observed, five among pulse-type populations and another one among burst-type populations. The statistical analysis shows that they can be easily differentiated suggesting that populations producing each pattern belong to a different cryptic species of the *L. longipalpis* complex. Further studies will be important to better understand the mechanism by which the song may be contributing to reproductive isolation.

## Additional files

Additional file 1:
**Sample of a train of a Pulse 1 song.**
Additional file 2:
**Sample of a train of a Pulse 2 song.**
Additional file 3:
**Sample of a train of a Pulse 3 song.**
Additional file 4:
**Sample of a train of a Pulse 4 song.**
Additional file 5:
**Sample of a train of a Pulse 5 song.**
Additional file 6:
**Sample of a train of a Burst song.**
Additional file 7:
**Table with the post-hoc results in the burst-type populations.**
