# Supplementary material for: Phenotypic differentiation in love song traits among sibling species of the Lutzomyia longipalpis complex in Brazil
Source: Parasit Vectors. 2015 May 28;8:290. doi: 10.1186/s13071-015-0900-8 (PMC4456791; doi:10.1186/s13071-015-0900-8)
Supplement: Additional file 7: — Table with the post-hoc results in the burst-type populations. [file 13071_2015_900_MOESM7_ESM.docx]

Supporting information 7: Table with the Post-Hoc results in the Burst-type populations.

|  | Sobral 2S | Natal | Marajó | Pancas | Jaíba 2S | Estrela 2S | Nova Porteirinha | Barcarena | Itamaracá | Passira |
| --- | --- | --- | --- | --- | --- | --- | --- | --- | --- | --- |
| Sobral 2S | # | # | # | # | # | # | # | # | # | # |
| Natal | 1.00 | # | # | # | # | # | # | # | # | # |
| Marajó | 1.00 | 1.00 | # | # | # | # | # | # | # | # |
| Pancas | 1.00 | 1.00 | 1.00 | # | # | # | # | # | # | # |
| Jaíba 2S | **0.04** | 0.06 | **0.02** | 0.15 | # | # | # | # | # | # |
| Estrela 2S | 1.00 | 1.00 | 1.00 | 1.00 | 0.97 | # | # | # | # | # |
| Nova Porteirinha | 1.00 | 1.00 | 0.78 | 1.00 | 1.00 | 1.00 | # | # | # | # |
| Barcarena | 0.51 | 0.78 | 0.23 | 1.00 | 1.00 | 1.00 | 1.00 | # | # | # |
| Itamaracá | 1.00 | 1.00 | 1.00 | 1.00 | 1.00 | 1.00 | 1.00 | 1.00 | # | # |
| Passira | 1.00 | 1.00 | 0.90 | 1.00 | 1.00 | 1.00 | 1.00 | 1.00 | 1.00 | # |

In bold are the significant values for the Post-Hoc pairwise comparison (Bonferroni).
